# Interplay between Non-Markovianity of Noise and Dynamics in Quantum Systems

**DOI:** 10.3390/e25030501

**Published:** 2023-03-14

**Authors:** Arzu Kurt

**Affiliations:** Department of Physics, Bolu Abant İzzet Baysal University, 14030 Bolu, Türkiye; arzukurt@ibu.edu.tr

**Keywords:** two-state system, non-Markovianity, continuous time random walk, non-Markovian noise

## Abstract

The non-Markovianity of open quantum system dynamics is often associated with the bidirectional interchange of information between the system and its environment, and it is thought to be a resource for various quantum information tasks. We have investigated the non-Markovianity of the dynamics of a two-state system driven by continuous time random walk-type noise, which can be Markovian or non-Markovian depending on its residence time distribution parameters. Exact analytical expressions for the distinguishability as well as the trace distance and entropy-based non-Markovianity measures are obtained and used to investigate the interplay between the non-Markovianity of the noise and that of dynamics. Our results show that, in many cases, the dynamics are also non-Markovian when the noise is non-Markovian. However, it is possible for Markovian noise to cause non-Markovian dynamics and for non-Markovian noise to cause Markovian dynamics but only for certain parameter values.

## 1. Introduction

Quantum non-Markovianity refers to the existence of memory effects in the dynamics of open quantum systems and has been the subject of many studies with the aim of defining, quantifying, and investigating various schemes to utilize it as a resource for quantum information tasks. Non-Markovianity has been discussed as a possible resource for quantum information tasks such as quantum system control [1], efficient entanglement distribution [2], perfect state transfer of mixed states [3], quantum channel capacity improvement [4], and efficiency of work extraction from the Otto cycle [5]. Miller et al. [6] carried out an optical study of the relation between non-Markovianity and the preservation of quantum coherence and correlations, which are essential resources for quantum metrology applications. Various approaches, from environmental engineering to classical driving to controlling the non-Markovianity of quantum dynamics, have been proposed, analyzed, and experimentally realized in recent years. Most non-Markovianity measures invoke a bidirectional exchange of information between the system and its environment at the root of the memory effects in the dynamics. The seeming contradiction between such an interpretation and the fact that even external classical noise could induce non-Markovian dynamics [7,8] was mostly resolved by showing that random mixing of unitary dynamics might lead to memory effects [9,10]. Representing the quantum environment of a finite-dimensional quantum system using classical stochastic fields has a long history. One of the drawbacks of such an approximation is the effective infinite temperature, which can be resolved by augmenting the master equation with extra terms to restore the correct thermal steady state. Another seemingly difficult task is to account for the lack of feedback from the system to the classical field. Despite these shortcomings, the stochastic Liouville equation (SLE) approach has produced various interesting physical models of open quantum systems [11,12,13,14,15,16].

There have been several studies on the effect of classical noise on the non-Markovianity of the quantum dynamics of two-state systems. For example, a study by Cialdi et al. investigated the relationship between different classical noises and the non-Markovianity of the dephasing dynamics of a two-level system [17]. The study found that non-Markovianity is influenced by the constituents defining the quantum renewal process, such as the time-continuous part of the dynamics, the type of jumps, and the waiting time. In addition, other studies have explored how to measure and control the transition from Markovian to non-Markovian dynamics in open quantum systems, as well as how to evaluate trace- and capacity-based non-Markovianity. It has been shown that classical environments that exhibit time-correlated random fluctuations can lead to non-Markovian quantum dynamics [18,19]. Costa-Filho et al. investigated the dynamics of a qubit that interacts with a bosonic bath and under the injection of classical stochastic colored noise [20]. The dynamic decoupling of qubits under Gaussian noise and RTN was investigated by Bergli et al. in [21,22]. Cai et al. showed that the environment being non-Markovian noise does not guarantee that the system’s dynamics are non-Markovian [23]. When the coupling of the bath to its thermalizing external environment is very strong or on time scales longer than the characteristic microscopic times of the bath, we expect that even fully quantum system-bath models reduce to this case [24]. The addition of non-equilibrium classical noise to dissipative quantum dynamics can be helpful in describing the influence of non-equilibrium environmental degrees of freedom on the transport properties [25]. Goychuk and Hanggi developed a method to average the dynamics of a two-state system driven by non-Markovian discrete noises of the continuous-time random walk type (multi-state renewal processes) [26].

The transition from Markovian to non-Markovian dynamics via tuning of the system-environmental coupling in various quantum systems has been reported [27,28,29,30,31]. The aim of the present study is to provide an answer to the question of whether there is any connection between the non-Markovianity of classical noise and the non-Markovianity of quantum dynamics of a two-state system (TSS) driven by such a noise source. Toward that end, we study the dynamics of a TSS driven by a continuous-time random walk (CTRW)-type stochastic process which is characterized by its residence time distribution (RTD) function. We investigate the effect of biexponential and manifest non-Markovian RTDs. The first one is a simple model of classical non-Markovian noise as a linear combination of two Markovian processes and allows one to study random mixing-induced quantum non-Markovianity, while the latter one can be tuned to study a large number of noise models. We find that exact analytical expressions for the trace distance and entropic measures of non-Markovianity of the dynamics can be obtained for a restricted set of system parameters. It is well known that Markovian classical noise can lead to non-Markovian quantum dynamics. Here, we show that when the driving noise is chosen to be expressively non-Markovian, one can still observe the Markovian quantum dynamics, depending on the noise and system parameters, albeit in a very restricted set. Hence, we show that the existence of non-Markovianity in classical noise does not guarantee quantum non-Markovianity of the dynamics of a TSS driven by that noise.

The outline of this paper is as follows. In Section 2, we describe the TSS and CTRW noise process and the noise averaging procedure that leads to the exact time evolution operator in the Laplace transform domain. The analytical and numerical results of the study for the biased and unbiased TSS for Markovian, as well as the non-Markovian CTRW process, are presented and discussed in Section 3. Section 4 concludes the article with a brief summary of the main findings.

## 2. Model and Non-Markovianity Measures

The main aim of this section is to introduce the TSS model which will be used to study the effect of the non-Markovianity of the classical noise on the non-Markovianity of the quantum dynamics of the TSS driven by the noise and to summarize the trace distance and entropy-based quantum non-Markovianity measures.

### 2.1. Model

We consider a two-state system (TSS) with the Hamiltonian

(1) $$H=\frac{1}{2}\hbar \epsilon_{0}\sigma_{z}+\frac{1}{2}\hbar \left(\Delta_{0}+\xi \left(t\right)\right)\sigma_{x}+\frac{1}{2}\left(E_{1}+E_{2}\right)I$$

where the $\sigma_{i}$ values are the Pauli operators, $E_{1,2}$ are the energies of states |1〉 and |2〉 of the TSS, $\Delta_{0}$ is the static tunneling matrix element, $\epsilon_{0}=\left(E_{2}-E_{1}\right)/\hbar$, and I is the identity operator. The TSS is driven by two-state non-Markovian noise with amplitudes $\xi \left(t\right)=\left\{\Delta_{+},\Delta_{-}\right\}$ and stationary-state probabilities $p_{\pm }^{st}=\langle \tau_{\pm }\rangle /\left(\langle \tau_{+}\rangle +\langle \tau_{-}\rangle \right)$, where $\langle \tau_{\pm }\rangle$ represents the average residence time of the noise in states $\Delta_{\pm }$. The stationary autocorrelation function of the noise is defined as $k\left(t\right)=\langle \delta \xi \left(t\right)\delta \xi \left(0\right)\rangle /\langle {\left[\delta \xi \right]}^{2}\rangle$, where $\delta \xi \left(t\right)=\xi \left(t\right)-{\langle \xi \rangle }_{st}$ and can be expressed in terms of the RTDs in the Laplace space as follows [25,26]:

(2) $$\begin{array}{ccc} k(s) & = & \frac{1}{s}-\left(\frac{1}{\langle \tau_{+}\rangle }+\frac{1}{\langle \tau_{-}\rangle }\right)\frac{1}{s^{2}}\frac{\left(1-\psi_{+}\left(s\right)\right)\left(1-\psi_{-}\left(s\right)\right)}{\left(1-\psi_{-}\left(s\right)\psi_{+}\left(s\right)\right)} \end{array}$$

where $\psi_{\pm }\left(s\right)$ are Laplace transforms of the residence time distribution of the noise in the $\Delta_{-}$ and $\Delta_{+}$ states and the autocorrelation time of the noise is defined using k(t) as $\tau_{\mathrm{corr}}=\int_{0}^{\infty }\left|k\left(t\right)\right|\;dt$. If k(t) is strictly positive for all *t*, then $\tau_{\mathrm{corr}}$ can be obtained from k(s) as $\tau_{\mathrm{corr}}=\lim_{s\to 0}k\left(s\right)$.

The dynamics of the density matrix ρ(t) of the TSS with the Hamiltonian in Equation (1) can be obtained by expressing it as $\rho \left(t\right)=\left[I+\sum_{i}P_{i}\left(t\right)\sigma_{i}\right]/2$, where $P_{i}\left(t\right)=\mathrm{Tr}\left[\rho \left(t\right)\sigma_{i}\right]$ is

(3) $$\dot{P}\left(t\right)=F\left(t\right)P\left(t\right)$$

where $P\left(t\right)={\left[P_{x}\left(t\right),P_{y}\left(t\right),P_{z}\left(t\right)\right]}^{T}$ and

(4) $$F\left[\xi \left(t\right)\right]=\left(\begin{array}{ccc} -\epsilon_{0} & 0 & 0\\ \epsilon_{0} & 0 & \xi (t)\\ 0 & \xi (t) & 0 \end{array}\right)$$

The noise propagator $S_{\pm }\left(t\right)=\exp\left(F[\Delta_{\pm }]\right)$ for the static values of noise $\xi =\left\{\Delta_{-},\Delta_{+}\right\}$ is

(5) $$S_{\pm }\left(t\right)=\sum_{k}R_{\pm }^{\left(k\right)}\exp\left(i\lambda_{\pm }^{\left(k\right)}t\right)$$

where $\lambda_{\pm }^{0}=0$, $\lambda_{\pm }^{1}=\Omega_{\pm }=\sqrt{\epsilon_{0}^{2}+\Delta_{\pm }^{2}}$, $\lambda_{\pm }^{2}=-\Omega_{\pm }$, and

(6) $$\begin{array}{ccc} R_{\pm }^{\left(0\right)} & = & \frac{1}{\Omega_{\pm }^{2}}\left(\begin{array}{ccc} \Delta_{\pm }^{2} & 0 & \epsilon_{0}\Delta_{\pm }\\ 0 & 0 & 0\\ \epsilon_{0}\Delta_{\pm } & 0 & \epsilon_{0}^{2} \end{array}\right)\\ R_{\pm }^{\left(1\right)} & = & {\left[R_{\pm }^{\left(2\right)}\right]}^{*}=\frac{1}{2}\left(\begin{array}{ccc} \frac{\epsilon_{0}^{2}}{\Omega_{\pm }^{2}} & i\frac{\epsilon_{0}}{\Omega_{\pm }} & -\frac{\epsilon_{0}\Delta_{\pm }}{\Omega_{\pm }^{2}}\\ i\frac{\epsilon_{0}}{\Omega_{\pm }} & 1 & i\frac{\Delta_{\pm }}{\Omega_{\pm }}\\ -\frac{\epsilon_{0}\Delta_{\pm }}{\Omega_{\pm }^{2}} & -i\frac{\Delta_{\pm }}{\Omega_{\pm }} & \frac{\Delta_{\pm }^{2}}{\Omega_{\pm }^{2}} \end{array}\right) \end{array}$$

The problem of obtaining the stationary noise average of the propagator in Equation (5) involves both averaging over the initial stationary probabilities. It was shown by Goychuk that this can also be performed exactly in the Laplace space for non-Markovian processes [25]. The noise-averaged propagator can be expressed as follows:

(7) $$\begin{array}{ll} S(s)= & p_{+}S_{+}\left(s\right)+p_{-}S_{-}\left(s\right)-\left(\frac{1}{\tau_{+}}+\frac{1}{\tau_{-}}\right)\left\{C_{+}+C_{-}\right.\\ & \left[A_{+}\left(s\right)B_{-}\left(s\right)+A_{-}\left(s\right)\right]{\left[I-B_{+}\left(s\right)B_{-}\left(s\right)\right]}^{-1}A_{+}\left(s\right)\\ & \left.\left[A_{-}\left(s\right)B_{+}\left(s\right)+A_{+}\left(s\right)\right]{\left[I-B_{-}\left(s\right)B_{+}\left(s\right)\right]}^{-1}A_{-}\left(s\right)\right\} \end{array}$$

where

(8) $$\begin{array}{ccc} S_{\pm }\left(s\right) & = & \sum_{k}\frac{R_{\pm }^{\left(k\right)}}{s-i\lambda_{\pm }^{\left(k\right)}}\\ A_{\pm }\left(s\right) & = & \sum_{k}R_{\pm }^{\left(k\right)}\frac{1-\psi_{\pm }\left(s-i\lambda_{\pm }^{\left(k\right)}\right)}{s-i\lambda_{\pm }^{\left(k\right)}}\\ B_{\pm }\left(s\right) & = & \sum_{k}R_{\pm }^{\left(k\right)}\psi_{\pm }\left(s-i\lambda_{\pm }^{\left(k\right)}\right)\\ C_{\pm }\left(s\right) & = & \sum_{k}R_{\pm }^{\left(k\right)}\frac{1-\psi_{\pm }\left(s-i\lambda_{\pm }^{\left(k\right)}\right)}{{\left(s-i\lambda_{\pm }^{\left(k\right)}\right)}^{2}} \end{array}$$

where ψ(s) is the Laplace transform of the distribution of the residence time of the noise.

### 2.2. Non-Markovianity Measures

Non-Markovianity of random processes has a well-established and widely accepted definition. The non-Markovianity of quantum dynamics, on the other hand, although the subject of an immense number of studies in recent years, has not reached a similar consensus. The trace distance-based measure of non-Markovianity developed in [32,33] quantifies the memory effect in the dynamics with the system’s retrieval of information from its environment, which shows up as nonmonotonic behavior in the distinguishability of quantum states. Given two density operators $\rho_{1}$ and $\rho_{2}$, the trace distance (TD) between them is defined as follows [34]:

(9) $$D\left(\rho_{1},\rho_{2}\right)=\mathrm{Tr}\sqrt{{\left(\rho_{1}-\rho_{2}\right)}^{†}\left(\rho_{1}-\rho_{2}\right)}$$

where Tr stands for the trace operation. TD is bounded from below by $D(\rho_{1},\rho_{2})=0$ for $\rho_{1}=\rho_{2}$ and from above by $D(\rho_{1},\rho_{2})=1$ if $\rho_{1}⊥\rho_{2}$. As a measure of distinguishability between two quantum states, it can be related to the probability of distinguishing two states with a single measurement [35].

Entropy-based Jensen–Shannon divergence (JSD) between two quantum states is another distinguishability measure used to quantify non-Markovianity [36,37] and is defined as the smoothed version of relative entropy:

(10) $$J\left(\rho_{1},\rho_{2}\right)=H\left(\frac{\rho_{1}+\rho_{2}}{2}\right)-\frac{1}{2}\left(H\left(\rho_{1}\right)+H\left(\rho_{2}\right)\right)$$

where H(.) is the von Neumann entropy H(ρ)=−Trρlogρ. $J(\rho_{1},\rho_{2})$ has the same bounds as the trace distance in the same limiting cases, but it is not a distance because, contrary to TD, it does not obey the triangle inequality. $\sqrt{J(\rho_{1},\rho_{2})}$ is shown to be a distance measure [38] and can be used to quantify the non-Markovianity of the quantum dynamics.

The non-Markovianity quantifiers based on a state distinguishability measure $D^{d}\left(\rho_{1},\rho_{2}\right)$ are defined as follows [32,33]:

(11) $$N^{d}=\max_{\rho_{1}\left(0\right),\rho_{2}\left(0\right)}\int_{\sigma_{d}\left(t\right)>0}\;\sigma_{d}\left(t\right)\;dt$$

where

(12) $$\sigma_{d}\left(t\right)=\frac{d}{dt}D^{d}\left(\rho_{1}\left(t\right),\rho_{2}\left(t\right)\right)$$

where the exponent *d* stands for either the trace distance distinguishability (*T*) or the Jensen–Shanon entropy divergence (*E*). Maximization in Equation (11) is carried out over all possible initial states $\rho_{1,2}\left(0\right)$. Wissmann et al. [39] showed that $\rho_{1}\left(0\right),\rho_{2}\left(0\right)$, chosen from the antipodal points of the Bloch sphere, maximizes the non-Markovianity measure based on the trace distance for two state systems [32,37]. For the problem studied, both the trace distance and Jensen–Shannon entropy divergence distinguishability measures could be expressed in terms of the population difference $P_{z}\left(t\right)$ and coherences $P_{x}\left(t\right)$ and $P_{y}\left(t\right)$ as follows:

(13) $$\begin{array}{lll} D^{T} & = & \sqrt{P_{x}^{2}+P_{y}^{2}+P_{z}^{2}} \end{array}$$

(14) $$\begin{array}{lll} D^{E} & = & \frac{1}{\sqrt{\log4}}\sqrt{2D^{T}\mathrm{arctanh}\left(D^{T}\right)+\log\left(1-{\left(D^{T}\right)}^{2}\right)} \end{array}$$

If the chosen distinguishability measure between any two initial states is a monotonic function of time, then the dynamics is said to be Markovian. Otherwise, $N^{d}$ quantifies the memory effects in the dynamics.

## 3. Results and Discussion

We first present the results for TSS, whose state energies were degenerated. When $\epsilon_{0}=0$, the Laplace transformed components of the evolution operator could be expressed in a simple form:

(15) $$\begin{array}{ccc} S_{yy}\left(s\right) & = & \frac{s\left(2s^{2}+\Delta_{-}^{2}+\Delta_{+}^{2}\right)}{2\left(s^{2}+\Delta_{-}^{2}\right)\left(s^{2}+\Delta_{+}^{2}\right)}+\frac{\Delta^{2}}{\tau }\left[\Psi \left(s\right)+\Psi^{*}\left(s\right)\right] \end{array}$$

(16) $$\begin{array}{ccc} S_{yz}\left(s\right) & = & -\frac{\Delta_{0}\left(s^{2}+\Delta_{-}\Delta_{+}\right)}{\left(s^{2}+\Delta_{-}^{2}\right)\left(s^{2}+\Delta_{+}^{2}\right)}-i\frac{\Delta^{2}}{\tau }\left[\Psi \left(s\right)-\Psi^{*}\left(s\right)\right] \end{array}$$

(17) $$\begin{array}{lll} S_{zz}\left(s\right) & = & S_{yy}\left(s\right),\;\;\;\;\;S_{zy}\left(s\right)=-S_{yz}\left(s\right) \end{array}$$

where

(18) $$\Psi \left(s\right)=\frac{\left[1-\psi \left(s+i\Delta_{-}\right)\right]\left[1-\psi \left(s+i\Delta_{+}\right)\right]}{{\left(s+i\Delta_{-}\right)}^{2}{\left(s+i\Delta_{+}\right)}^{2}\left[1-\psi \left(s+i\Delta_{-}\right)\psi \left(s+i\Delta_{+}\right)\right]}$$

We considered a symmetric two-state discrete noise process such that $\Delta_{+}=\Delta =-\Delta_{-}$ was the amplitude, $\tau_{+}=\tau_{-}=\tau$ was the mean residence time, and $\psi \left(s\right)=\psi_{+}\left(s\right)=\psi_{-}\left(s\right)$ was the residence time distribution function of the noise. Since one of the aims of the study was to investigate the relation between the non-Markovianity of the driver noise and the quantum dynamics it created, for the residence time distribution of the noise, we considered two non-Markovian models, namely the bi-exponential and manifest non-Markovian models, which have Markovian-limiting cases.

### 3.1. Markovian Noise

First, we considered the Markovian noise case, having an RTD ψ(s)=1/(1+sτ) which can be obtained with θ=0,1 for the limit of noise with a biexponentially distributed residence time (Equation (27)) or $t_{d}\to 0$ for the limit of the manifest non-Markovian RTD (Equation (31)), both of which are discussed in Section 3.2 and Section 3.3, respectively. For such an RTD, the inverse Laplace transform of the noise propagators in Equations (15)–(17), can be performed exactly to obtain the following:

(19) $$\begin{array}{ccc} P_{y}\left(t\right) & = & S\left(t\right)\;\sin\left(\Delta_{0}t+\phi \right) \end{array}$$

(20) $$\begin{array}{ccc} P_{z}\left(t\right) & = & S\left(t\right)\;\cos\left(\Delta_{0}t+\phi \right) \end{array}$$

where the initial values of $P_{y}\left(t\right)$ and $P_{z}\left(t\right)$ are parameterized in terms of ϕ as $P_{y}\left(0\right)=\sin\phi$ and $P_{z}\left(0\right)=\cos\phi$. S(t) in Equations (19) and (20) is the stochastic evolution operator of the Markovian two-state noise:

(21) $$S\left(t\right)=e^{-t/\tau }\;\left[\cosh\left(\sqrt{1-\Delta^{2}\tau^{2}}\;t\right)+\frac{1}{\sqrt{1-\Delta^{2}\tau^{2}}}\sinh\left(\sqrt{1-\Delta^{2}\tau^{2}}\;t\right)\right]$$

The trace distance distinguishability of the dynamics can be calculated with Equation (13) by inserting the population and coherence expressions from Equations (19) and (20) as follows:

(22) $$D\left(\rho_{1},\rho_{2}\right)=\left|S(t)\right|$$

One should note that S(t) is a monotonously decreasing function of *t* for Δτ<1 but displays decaying oscillations when Δτ>1 as the hyperbolic trigonometric functions inside the parentheses transform to ordinary trigonometric functions when Δτ>1. The non-Markovianity measure (Equations (11) and (12)) is defined as the integral of the positive values of the time derivative of D, N=0 for Δτ<1. Interestingly, the trace distance distinguishability-based non-Markovianity measure for this particular D and Δτ>1 can be obtained analytically in a simple form as follows:

(23) $$N=\frac{1}{e^{\frac{\pi }{\sqrt{\Delta^{2}\tau^{2}-1}}}-1}$$

Here, the non-Markovianity is found to be independent of the static value of the coupling coefficient $\Delta_{0}$. A similar expression for N was reported in [18] for a similar Markovian two-state noise. It is also easy to obtain an analytical expression for the Jensen–Shannon entropy divergence for the present case as follows:

(24) $$J\left(t\right)=\frac{1}{\log4}\left\{\log\left[1-S^{2}\left(t\right)\right]+2S\left(t\right)\mathrm{arctanh}\left[S(t)\right]\right\}$$

**Figure 1:**
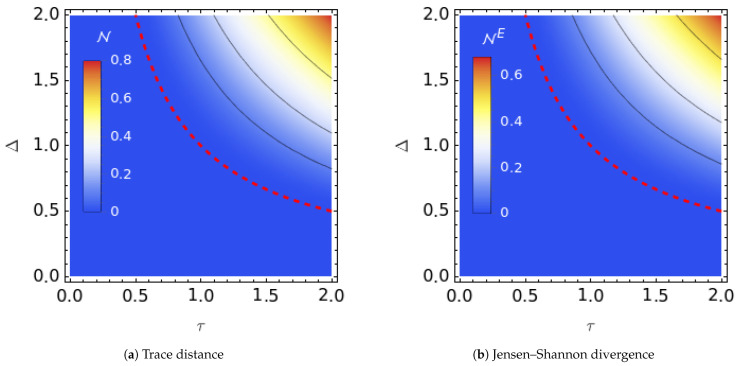
Non-Markovianity of the dynamics for the unbiased TSS as a function of the Markovian noise with an auto-correlation time τ and the amplitude Δ based on the trace distance (**a**) and Jensen–Shannon divergence distinguishability (**b**). The red dotted line is the zero contour, while the straight lines denote N equal to 0.1, 0.25, and 0.5.

Although it is possible to derive an exact expression for an entropy-based non-Markovianity measure by using Equations (11) and (24), the expression is not compact enough to be helpful in deciphering the relation between $N^{E}$ and the noise parameters. Therefore, we display only the calculated entropy-based $N^{E}$ along with the one derived from the trace distance distinguishability in Figure 1.

The contours of non-Markovianity are plotted in Figure 1 as functions of the mean residence time τ and noise amplitude Δ. As can be seen in Equation (23) and the plot, N is nonzero as long as the Kubo number of the noise is greater than one, which is known as a slow noise, strong system noise coupling, or strongly colored noise regime [40] Interestingly, both measures were found to signal the same limits (Δτ>1) for the existence of non-Markovianity in the dynamics. Furthermore, even the magnitudes of N and $N^{E}$ were found to be comparable. We observed the same behavior for all the other noise models reported in the following, and for the remainder of the paper, we will report the results only for the trace distance-based measure N.

An interesting dynamics and non-Markovianity behavior was observed if the noise RTD was chosen to have the α→0 limit of the manifest non-Markovian RTD in Equation (31), which reduced ψ(s) to a form similar to that of Markovian noise with a modified mean residence time. It is easy to perform an exact analytical inverse Laplace transform of the propagator expressions in Equations (15)–(17) for ψ(s)=1/(1+sτtanh(1)) and find the population difference as follows:

(25) $$P_{z}\left(t\right)=\frac{1}{1+e^{2}}\left(2\cos\left(\Delta t\right)+\left(e^{2}-1\right)S_{2}\left(t\right)\right)$$

where

(26) $$S_{2}\left(t\right)=e^{-ct/\tau }\left(\cosh\left(tC/\tau \right)+\frac{1+e^{2}}{\sqrt{{\left(1+e^{2}\right)}^{2}-{\left(e^{2}-1\right)}^{2}\Delta^{2}\tau^{2}}}\sinh\left(tC/\tau \right)\right)$$

where $C=\sqrt{\coth^{2}1-\Delta^{2}\tau^{2}}$ and c=coth1. As *t* approaches infinity, $S_{2}\left(t\right)$ approaches zero, while $P_{z}\left(t\right)$ exhibits oscillations with an amplitude of $2/(1+e^{2})$ and a frequency of Δ. The non-Markovianity of the dynamics, as assessed by both the trace distance and Jensen–Shannon entropy, was found to be unbounded. It is worth noting that the long-term limit of $P_{z}\left(t\right)$ was insensitive to both the noise amplitude Δ and the mean residence time τ. This result contradicts the findings obtained for Markovian noise, for which we found that N is zero for Δτ<1 and tends toward a finite value for Δτ>1. It should be noted that the α→0 limit of a manifest non-Markovian process describes a noise with 1/ω as the power spectrum [41] near ω=0, which is similar to the widely studied 1/f noise. Benedetti et al. studied [18] the non-Markovianity of colored $1/f^{\alpha }$ noise-driven quantum systems and reported finite values for N, in contrast to our findings.

### 3.2. Biexponentially Distributed Residence Time

The biexponential RTD in the time domain is defined as follows [41]:

(27) $$\psi \left(t\right)=\theta \alpha_{1}\exp\left(-\alpha_{1}t\right)+\left(1-\theta \right)\alpha_{2}\exp\left(-\alpha_{2}t\right)$$

where θ and (1−θ) are the probabilities of the realization of the transition rates $\alpha_{1}$ and $\alpha_{2}$, respectively. The mean residence and autocorrelation times of this noise can be expressed as follows:

(28) $$\begin{array}{ccc} \langle \tau \rangle & = & \theta /\alpha_{1}+\left(1-\theta \right)/\alpha_{2} \end{array}$$

(29) $$\begin{array}{ccc} \tau_{\mathrm{corr}} & = & \int_{0}^{\infty }\left|k\left(t\right)\right|\;dt \end{array}$$

where θ=0 and θ=1 correspond to Markovian noise with mean residence times $1/\alpha_{1}$ and $1/\alpha_{2}$, respectively. The two-state noise with biexponential residence time distribution allows one to define a non-Markovianity quantifier, denoted by $C_{V}$, which can be tailored by tuning the parameter θ. This quantifier is given by the ratio of the mean autocorrelation time of the non-Markovian noise, $\langle \tau_{\mathrm{corr}}\rangle =\int_{0}^{\infty }k\left(t\right)dt$, to the autocorrelation time of the Markovian process $\tau_{\mathrm{corr}}^{M}=\langle \tau \rangle /2$ through the mean residence time 〈τ〉 as in Equation (30):

(30) $$C_{V}^{2}=\frac{2}{\langle \tau \rangle }\tau_{corr}$$

The Laplace-transformed expressions for the noise propagator in Equations (15)–(17) for the biexponential RTD are amenable to be transformed back to the time domain for the unbiased TSS. However, the resulting population, coherence, and trace distance expressions are tedious to display here. On the other hand, for the manifest non-Markovian RTD, the only way to perform the inverse transformation is to use numerically exact inverse Laplace transformation (ILT) methods. We tested the CME [42], Crump [43], Durbin [44], Papoulis [45], Piessens [46], Stehfest [47], Talbot [48], and Weeks numerical ILT algorithms and found that the method based on concentrated matrix exponential (CME) distributions reported in [42] had the best performance in terms of computational cost for a given accuracy. The convergence of the computed quantities as a function of the number of included terms and the working precision was carefully checked, and 300 terms and 64 bit precision were found to be adequate for all the reported calculations to converge to 0.1%.

**Figure 2:**
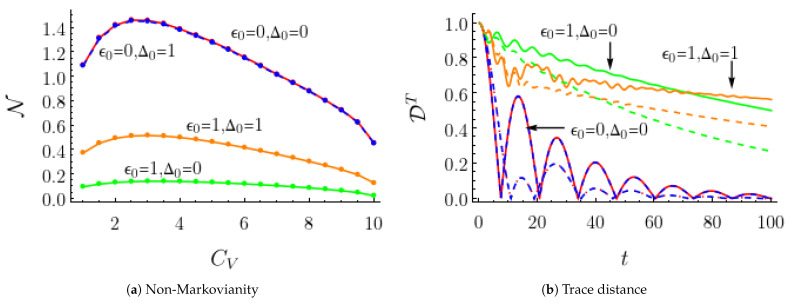
Noise non-Markovianity $C_{V}$’s dependence on the trace-distance based non-Markovianity measure N (**a**) and trace distance distinguishability $D^{T}$ (**b**) for the two-state discrete noise with bi-exponential residence time distribution. The noise parameters were Δ=1/4, $\alpha_{1}=1/20$, and $\alpha_{2}=1$. θ values were chosen such that $C_{V}$ ranged from 1 to 10. N and $D^{T}$ for four combinations of TSS transition energy $\epsilon_{0}$ and electronic coupling $\Delta_{0}$ values are displayed. Note that for the unbiased case ($\epsilon_{0}=0$), the difference in N between $\Delta_{0}=0$ and $\Delta_{0}=1$ is minimal and indistinguishable on the plots. The straight (dashed) lines in $D^{T}$ plots of (**b**) were calculated at $C_{V}=4$ (10).

N of the TSS dynamics as a function of the noise non-Markovianity parameters $C_{V}$ is shown in Figure 2a for a noise amplitude Δ=1/4 with $\Delta_{0}=0,1$ and $\epsilon_{0}=0,1$. Remarkably, it was observed that for the four combinations of the site energy difference $\epsilon_{0}$ and the static coupling $\Delta_{0}$, the non-Markovianity of the quantum dynamics displayed a broad resonance structure as a function of $C_{V}$, which indicates that increasing the non-Markovianity of the classical driving noise beyond a certain threshold would decrease the non-Markovianity of the driven quantum dynamics. Figure 2b shows the trace distance distinguishability at two chosen $C_{V}$ values and indicates that the main effect of increasing $C_{V}$ is to increase the dissipation rate of the dynamics. These results indicate that the increasing non-Markovian nature of the driving noise might increase, but it might also decrease the non-Markovianity of the quantum dynamics of the system studied, depending on the magnitude.

### 3.3. The Manifest Non-Markovian Noise

The other residence time distribution we will investigate is a manifest non-Markovian noise with the RTD defined in the Laplace space as follows [26,41]:

(31) $$\psi \left(s\right)=\frac{1}{1+s\tau g(s)}$$

with

(32) $$g\left(s\right)=\frac{\tanh\left[{\left(st_{d}\right)}^{\alpha /2}\right]}{{\left(st_{d}\right)}^{\alpha /2}}$$

where τ is the mean residence time of the noise and $t_{d}$ is another time constant that can be used to control the non-Markovianity of the noise. (At the limit $t_{d}$ = 0, ψ(t) is exponential). The parameter α, which is limited to the range 0<α<1, characterizes the noise-power distribution, where ψ(s) describes noise that shows $1/\omega^{1-\alpha }$ features in its spectrum as ω→0 and encompasses various power-law residence time distributions. α=1 describes normal diffusion, while the 0<α<1 case corresponds to subdiffusion with an index α in the transport context [41]. One of the interesting properties of discrete, manifestly non-Markovian noise is that its correlation time is infinite for α<1, which means that the Kubo number is effectively infinite, and no perturbative treatment would produce any reasonably accurate dynamics. The current method based on the Laplace transform is the only way to investigate the dynamics for such residence time distributions. We discussed the two limiting cases, namely $t_{d}\to 0$ (Markovian) and α→0 (infinite C), of the manifest non-Markovian RTD above. Here, we present and discuss how the RTD parameters α and $t_{d}$ affect the trace distance distinguishability and non-Markovianity of the TSS dynamics with different system parameters.

**Figure 3:**
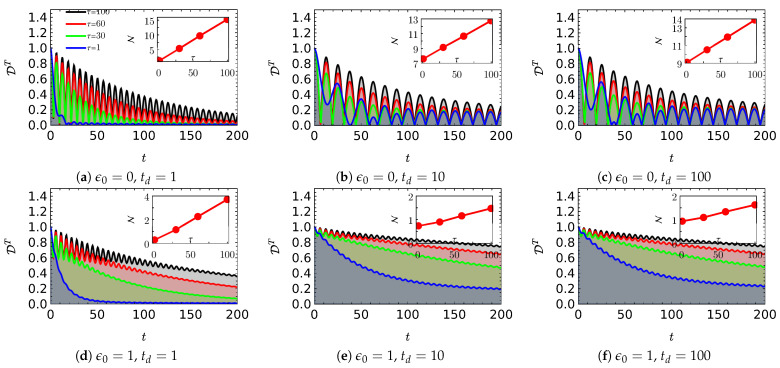
Trace distance as a function of time for the manifestly non-Markovian noise at different $t_{d}$ parameters and average residence times τ. Insets show the trace distance-based non-Markovianity measure as a function of τ. The other parameters of the noise and the system are α=1/2, $\Delta_{0}=0$, and Δ=1/2.

First, we present the trace distance distinguishability along with the associated non-Markovianity N for the manifestly non-Markovian noise for various $t_{d}$ and mean residence time τ values in Figure 3 for a biased and unbiased TSS at α=0.5 and Δ=0.5. As $t_{d}$ is a rough measure of the non-Markovianity of manifest non-Markovian noise, one can infer, from a comparison of the insets in Figure 3a,c as well as Figure 3c,d, that N increases with an increasing $t_{d}$ for both the unbiased and biased TSS. The mean residence time dependence of N was found to be independent of $t_{d}$. N increased with an increasing τ for all three values considered in this work for the biased as well as the unbiased TSS. Furthermore, N in the biased case is always found to be lower than that of the unbiased case. Another interesting observation from Figure 3b is that the trace distance distinguishability for the TSS driven by the highly non-Markovian noise tended toward a nonzero constant instead of the expected zero value.

**Figure 4:**
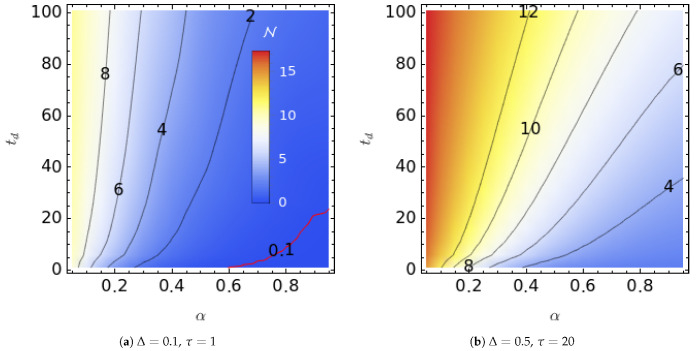
Dependence of α and $t_{d}$ of trace distance-based non-Markovianity N on the dynamics of TSS driven with manifest non-Markovian two-state noise at different Kubo numbers: K=0.1 (**a**) and K=10 (**b**). The same color map is used for both plots, and the iso-N values are shown as the contour labels. The red contour line in (**a**) is the N=0.1 contour.

To further delineate the relationship between N and the noise parameters α and $t_{d}$, we present the trace distance-based non-Markovianity measure N as a function of the exponent α and the $t_{d}$ time parameter of the noise residence time distribution for the dynamics of the unbiased TSS in Figure 4 in two different combinations of noise amplitude and mean residence time. The mean residence time of the noise is τ=1,20 in these graphs, and the amplitude of the noise chosen is Δ=0.1,0.5 for the subgraphs. The most important observation from Figure 4 is that the Kubo number was the most important noise parameter that determined the magnitude of the non-Markovianity of the TSS dynamics. The larger Δ led to a larger N for given α and $t_{d}$ values. This finding is similar to the one we discussed above for Markovian noise; the existence of non-Markovianity in that case depended on if Δτ>1. For the manifest non-Markovian noise, the dynamics were found to be non-Markovian even for Δτ<1. However, the magnitude of N still strongly depended on the Kubo number K=Δτ. Figure 4 also indicates that N depends on $t_{d}$ weakly above a threshold (around $t_{d}=15$), and N increases smoothly with α for a constant $t_{d}$ in most of the $\alpha -t_{d}$ plane. It should also be noted that N can be zero under manifest non-Markovian noise driving as α→1 when Δ≪1. This limit corresponds to white noise with a constant power spectrum at all frequencies.

## 4. Conclusions

We studied Jensen–Shannon entropy divergence and trace distance-based measures of non-Markovianity of the dynamics of a two-level system under continuous-time random walk-type stochastic processes with Markovian and non-Markovian residence time distributions to delineate whether there was any connection between the Markovianity of the noise and that of the dynamics. We were able to obtain analytically exact expressions for both measures for the unbiased TSS driven by Markovian CTRW noise. This expression indicates that, above a critical Kubo number for the noise, even Markovian noise can lead to non-Markovian quantum dynamics. The numerical study of a biased TSS with the same external noise was found to be mainly a smearing of the exact boundary between the Markovian and non-Markovian boundary in the noise frequency-noise amplitude or the classical noise-TSS coupling coefficient plane. We used non-Markovian noise with a biexponential distribution as a model of the non-Markovianity produced by random mixing of Markovian dynamics and found that increasing the non-Markovianity of the noise might not lead to increased N values for the dynamics. We also considered a CTRW with a manifest non-Markovian residence time distribution and showed that the dynamics can be Markovian even for such noise. An interesting finding of this study was obtained at the α→0 limit of manifest non-Markovian noise. The exact expression obtained for the trace distance at this limit showed that N was infinite at this limit. As the discussion on the proper definition and measure of the non-Markovianity of quantum dynamics has not been settled yet, the results reported in this study provide a case study for answering the question “does the non-Markovianity of the classical driver determine the non-Markovianity of the driven”?

## Data Availability

Data are available from the author upon reasonable request.
